# A Retrospective Comparison of Ultrasound-Assisted Catheter-Directed Thrombolysis and Catheter-Directed Thrombolysis Alone for Treatment of Proximal Deep Vein Thrombosis

**DOI:** 10.1007/s00270-016-1367-5

**Published:** 2016-06-01

**Authors:** Vladimir Y. I. G. Tichelaar, Ellen E. Brodin, Anders Vik, Trond Isaksen, Finn Egil Skjeldestad, Satish Kumar, Nora C. Trasti, Kulbir Singh, John-Bjarne Hansen

**Affiliations:** K. G. Jebsen – Thrombosis Research and Expertise Centre (TREC), Department of Clinical Medicine, UiT – The Arctic University of Norway, 9037 Tromsø, Norway; Division of Hemostasis and Thrombosis, Department of Hematology, University Medical Center Groningen, Groningen, The Netherlands; Division of Internal Medicine, University Hospital of North Norway, Tromsø, Norway; Research Group Epidemiology of Chronic Diseases, Department of Community Medicine, UiT – The Arctic University of Norway, 9037 Tromsø, Norway; Department of Radiology, University Hospital of North Norway, Tromsø, Norway

**Keywords:** Catheter, Thrombolysis, Ultrasound, Venous thrombosis, Quality of life, Post-thrombotic syndrome

## Abstract

**Background:**

Recent studies have suggested that catheter-directed thrombolysis (CDT) reduces development of post-thrombotic syndrome (PTS). Ultrasound-assisted CDT (USCDT) might enhance the efficiency of thrombolysis. We aimed to compare USCDT with CDT on efficacy, safety, development of PTS, and quality of life after long-term follow-up.

**Methods:**

We describe a retrospective case series of 94 consecutive patients admitted with iliofemoral or more proximal deep vein thrombosis (DVT) to the University Hospital from 2002 to 2011, treated either with CDT or USCDT. Scheduled follow-up visits took place between April 2013 and January 2014. Venography measured the degree of residual luminal obstruction of the affected veins. Each patient completed the Short Form 36-item health survey assessment and the Venous Insufficiency Epidemiological and Economic Study-Quality of Life/Symptoms questionnaires. PTS was assessed using the Villalta scale.

**Results:**

Risk factors of DVT were equally distributed between groups. In the USCDT group, we observed a significant decline in the duration of thrombolytic treatment (<48 h: 27 vs. 10 %), shortened hospital stay (median 6.0 days (IQR 5.0–9.0) vs. 8.0 (IQR 5.8–12.0)), and less implantation of (intravenous) stents (30 vs. 55 %). There was no difference in patency (76 vs. 79 % fully patent), prevalence of PTS (52 vs. 55 %), or quality of life between groups after long-term follow-up (median 65 months, range: 15–141).

**Conclusions:**

In this observational study, USCDT was associated with shortened treatment duration, shorter hospital stay, and less intravenous stenting, compared to CDT alone without affecting the long-term prevalence of PTS or quality of life.

## Introduction

The post-thrombotic syndrome (PTS) causes considerable morbidity in patients after deep vein thrombosis (DVT) of the lower extremity. After 2 years, 30–50 % of the patients will develop PTS, of which 10 % will have moderate and 3–5 % severe PTS [1, 2]. Risk factors for (severe) PTS are thrombus proximity and recurrent ipsilateral venous thrombosis [1, 3] where prevention of the latter is the only effective way to prevent the increased severity and frequency of PTS [4]. The wearing of compression stockings, still recommended by leading guidelines [5], has recently been questioned [6].

Recent studies have shown that catheter-directed thrombolysis (CDT) reduces the development of PTS. The CAVENT study reported 14.4 % [95 % confidence interval (CI) 0.2–27.9] and 28 % (95 % CI 14–42) absolute risk reduction of PTS after 2 and 5 years of follow-up, respectively, when CDT was compared with standard treatment (anticoagulation and compression stockings) [7, 8]. A recent Cochrane review on CDT reported a relative reduction of 25 % of PTS [9]. As PTS is more common in patients with a proximal DVT [1, 10], guidelines recommend the use of CDT in selected patients with iliofemoral venous thrombosis [5, 11]. However, a recent cross-sectional study of patients with proximal lower-extremity DVT showed that CDT was resource-demanding and increased therapy-related adverse events [12]. Unfortunately, this study did not comprise outcomes such as recurrent venous thrombosis, late mortality, and incidence of PTS in the analyses [12].

Ultrasound-assisted CDT (USCDT), combining CDT with a catheter system that uses high frequency ultrasound, might enhance the efficiency of the thrombolytic process even more. In a case series of 53 patients, treatment with USCDT led to reduced total infusion time of the thrombolytic agent, a greater incidence of complete clot lysis, and a reduction in bleeding rates, compared with historical data [13]. In vitro data indicate that ultrasound facilitates thrombolysis by making more plasminogen receptor sites available for the thrombolytic agent [14]. A single center experience suggests that USCDT may be an equally safe and efficacious treatment for DVT as CDT alone [15]. Only one study has previously conducted a direct comparison of USCDT and CDT for the treatment of acute iliofemoral DVT of lower extremity [16]. Forty-eight patients were randomized between CDT and USCDT with a fixed-dose alteplase (20 mg/15 h). The primary outcome of residual thrombus load after 15 h of treatment did not differ between treatment modalities nor did the bleeding rate and quality of life, 3 months after therapy [16].

The incidence and severity of PTS among DVT patients tend to increase during the first 2 years after the DVT [1], and the CAVENT study showed that follow-up had to exceed 6 months in order to detect a benefit of CDT on the incidence of PTS [7]. Therefore, it is likely assumed that it will take more than 3 months to be able to observe differential impact of USCDT versus CDT on development of PTS. We aimed to compare USCDT with CDT in a case series of patients with DVT on efficacy, safety, degree of PTS, and quality of life after long-term (>12 months) follow-up.

## Methods

Our study is a retrospective case series of consecutive patients admitted to the University Hospital from January 2002 to January 2012 with iliofemoral or vena cava inferior DVT, who were treated with CDT or USCDT. From 2009 on, the patients were preferentially treated with USCDT (85 % of all cases treated after 2009). Due to random lack of US catheters, a few patients were treated with CDT only. Our design thus closely resembles a historical cohort study. Inclusion criteria were an objectively diagnosed DVT using ultrasound examination (64 %) or venography (36 %), extending into vena iliaca externa or vena cava inferior. Exclusion criteria for any catheter-directed thrombolysis were prior cerebral bleeding, a thrombotic or embolic cerebral infarction in the last 3 months, head trauma or major surgery in the last 14 days, bleeding tendency, platelet counts below 100 × 10^9^/L, pregnancy, hypertension (systolic blood pressure above 180 mmHg or diastolic blood pressure above 110 mmHg), renal failure, liver failure, and malignant disease with an expected survival of less than 1 year [17]. All patients alive as on April 1, 2013 were eligible for a follow-up visit at the hospital between April 2013 and January 2014, to clinically evaluate the effects of treatment. As it is the obligation of physicians to monitor the efficacy and safety of introduction of new treatment modalities, a review by the Institutional Review Board was not performed. Informed consent was obtained from all the participants included in this study.

All patients were treated with low molecular weight heparin (LMWH) directly after diagnosis of DVT and they underwent an initial venography, followed by thrombolytic therapy. Interventions were patency-driven, and venography was performed at intervals of 24 h. The treatment protocols of CDT and USCDT, including inclusion/exclusion criteria, type and dosage scheme of thrombolytic agent, and evaluations during treatment remained unchanged over the time period from 2002 to 2013, except for the catheters used. The responsible senior consultants at the radiology and hematology departments were experienced physicians and remained unchanged over the time period.

In the USCDT group, the EKOS EkoSonic^®^ Endovascular System with MACH 4e (EKOS Corporation, 11911 South Bothell, WA 98011, USA) was used [18]. Frequency of the ultrasound was 2.05–2.35 MHz with a power of 50.0 W pulse power maximum (30.0 W average) resulting in 0.5 W per ultrasound transducer unit. In the CDT group, a catheter with multiple side holes (UNI-FUSE, AngioDynamics or Cragg-McNamara^®^ Valved Infusion Catheter) was used.

Venous access was obtained through popliteal vein in both groups. Balloon dilatation and deflation were performed when deemed necessary while evaluating treatment result. Self-expandable nitinol stents ranging in diameter from 10 to 16 mm were used when deemed necessary by the treating radiologist and hematologist (organized thrombus, clear signs of May–Thurner syndrome or when other external compression was suspected (e-Luminex, Bard; Protégé, EV3 or Sinus-XL stent, Optimed)). These criteria did not change during the study period.

A bolus of 5000 IE unfractionated heparin (UFH) and 5 mg rt-PA (Actilyse; Boehringer Ingelheim, GmbH) was injected through the catheter at the start of both catheter-directed thrombolysis procedures, followed by continuous infusion of rt-PA, 0.01 mg/kg/h, and UFH, 300 IU/kg/24 h. The activated partial thromboplastin time (normal range 25–36 s) was measured twice daily and 6 h after adjustment of the UFH dose to keep the APTT between 50 and 70 s during treatment. Plasma fibrinogen was measured twice daily, and if fibrinogen was reduced to or below 1.0 g/L, the infusion of rt-PA was stopped for 2 h and then restarted at 50 % of the original dose. Hemoglobin, platelet count, PT-INR, and d-dimer were measured once daily during treatment. Blood pressure and heart rate were monitored, and inspection of the leg was performed regularly.

After both thrombolytic procedures, anticoagulant therapy proceeded with standard DVT treatment. According to our treatment guidelines, oral anticoagulant treatment with warfarin was recommended for 1 year or indefinitely in patients with stent implantation, with a treatment intensity of 2.0–3.0 PT-INR.

Clot burden was categorized into three groups (length of <10, 10–30, and >30 cm). The degree of thrombolysis after the procedure was visualized by venography and categorized into no effect or progression, less than 50 % lysis (grade I), 50–90 % lysis (grade II), and more than 90 % lysis (grade III). Percentage clot lysis was estimated by the difference in the length of thrombus before versus after treatment. Major bleeding was defined as any clinically overt bleeding that resulted in the cessation of therapy, further hospitalization, death or that required transfusion or surgical intervention. All other bleedings were classified as minor. Lowering of the standard dosing regimen of rt-PA according to the plasma fibrinogen level (see paragraph above) was defined as the outcome ‘less thrombolytic dose.’

At follow-up, a new venography was performed to measure the degree of residual luminal obstruction. Patency were defined as open when there was no vessel stenosis and no collateral venous drainage, as stenosis when an open venous segment had significant stenosis (50–90 %) or in case of severe vessel stenosis (>90 %) with collateral venous drainage, and as occluded when total occlusion of a venous segment with collateral venous drainage was observed.

The diagnosis of post-thrombotic syndrome (PTS) was made using the Villalta Scale [19]. PTS is categorized by severity: mild (≥ 5 < 10 points), moderate (≥ 10 < 15 points), and severe (≥ 15 points). The presence of venous ulcers directly accrues 15 points on the Villalta Scale [20]. To assess quality of life, we included the Short Form Health Survey-36 (SF-36) [21] and the Venous Insufficiency Epidemiological and Economic Study-Quality of Life/Symptoms (VEINES-QOL/Sym) questionnaires [22].

Data were analyzed using IBM SPSS Statistics, version 22 (Armonk, NY, United States of America). Differences in categorical data between treatment modalities were analyzed using Pearson’s Chi-squared test, and differences in continuous data were analyzed with Student’s *t*-test or Mann–Whitney *U*-test when data were not normally distributed. The maximum percentage of missing data for a given variable was 11.8 % within the VEINES or SF-36 questionnaire, and appeared to have a random pattern. Values are expressed as means ± one standard deviation if data were normally distributed and as medians with 25 and 75th percentiles in parenthesis (IQR) if data were not normally distributed.

## Results

We included 94 patients with an iliofemoral or inferior vena cava DVT, accounting for 95 events since one patient experienced a second DVT. Sixty-two patients were treated with CDT (6 of these after 2009) and 33 with USCDT. Characteristics of patients, risk factors for DVT, location of thrombi, and duration of symptoms, stratified by treatment modality are shown in Table 1. There were no significant differences between groups.

**Table 1:** Baseline characteristics of 94 patients with a proximal DVT of the leg, treated with (ultrasound-assisted) catheter-directed thrombolysis

	CDT (*n* = 62)	USCDT (*n* = 33)^a^
	% (*n*) or median (IQR)	% (*n*) or median (IQR)
Age (years)	49.5 (34.0–62.3)	34.0 (21.5–57.0)
Men	36 (22)	21 (7)
Provoking risk factors		
Surgery	10 (6)	12 (4)
Trauma	7 (4)	9 (3)
Immobilization	3 (2)	0 (0)
Cancer	11 (7)	9 (3)
Pregnancy	0 (0)	0 (0)
Puerperium	8 (3)	12 (3)
Estrogens	33 (13)	23 (6)
Other risk factors		
Obesity	20 (12)	36 (12)
Thrombophilia	5 (3)	9 (3)
Previous VTE	16 (10)	21 (7)
Acute non-surgical illness	21 (13)	12 (4)
Unprovoked	52 (32)	55 (18)
With pulmonary embolism	19 (12)	21 (7)
Location of DVT		
Vena cava inferior	27 (17)	33 (11)
V. iliaca ext./communis	72 (45)	67 (22)
Duration of symptoms (days)	4.0 (1.0–7.0)	3.0 (1.0–9.0)

*CDT* catheter-directed thrombolysis, *USCDT* ultrasound-assisted CDT, *IQR* interquartile range (25–75th percentile), *VTE* venous thromboembolic event, *DVT* deep vein thrombosis

^a^No significant differences between groups

Table 2 displays the characteristics of venous thrombus and short-term outcomes after treatment. The thrombus burden was equal in both groups before the start of treatment. The proportion of patients that achieved sufficient patency within 48 h was significantly higher after USCDT (27 vs. 10 %, *P* < 0.05). The median duration of total hospitalization was shorter for USCDT patients compared to those treated with CDT [6.0 days (IQR 5.0–9.0) vs. 8.0 (IQR 5.8–12.0), *P* < 0.05]. Finally, intravenous stenting of residual thrombosis after thrombolysis was less often deemed necessary by the treating team in the USCDT group (30 % vs. 55 %, *P* < 0.05). We did not observe any significant differences in the dose of thrombolytic agent used, nor in the degree of thrombolysis achieved immediately after treatment [>90 % of luminal recanalization in 88 % (CDT) vs. 76 % (USCDT)]. Exclusion of patients (*n* = 8) with symptoms of VTE longer than 14 days, an exclusion criterion in most guidelines [5, 11], did not change outcomes (data not shown).

**Table 2:** Characteristics of venous thrombus and short-term outcomes after (ultrasound-assisted) catheter-directed thrombolysis in 95 cases of iliofemoral or inferior vena cava deep vein thrombosis

	CDT (*n* = 62)	USCDT (*n* = 33)
	% (*n*) or median (IQR)	% (*n*) or median (IQR)
Length of thrombus		
< 10 cm	0 (0)	0 (0)
10–30 cm	25 (15)	27 (9)
> 30 cm	75 (46)	73 (24)
Duration of intervention (hours)		
< 48	10 (6)	27 (9)*
49–72	41 (26)	33 (11)
72–119	38 (23)	25 (8)
> 120	11 (7)	15 (5)
Additional intervention		
Balloon dilatation	92 (57)	94 (31)
Stenting	55 (34)	30 (10)*
Less thrombolytic dose	21 (13)	27 (9)
Degree of thrombolysis		
No effect or progression	2 (1)	0 (0)
Grade I (< 50 %)	0 (0)	0 (0)
Grade II (50–90 %)	10 (6)	24 (8)
Grade III (> 90 %)	88 (54)	76 (25)
Hospitalization time (days)	8.0 (5.8–12.0)	6.0 (5.0-9.0)*
Safety outcomes		
Death by bleeding	0 (0)	0 (0)
Minor bleeding	21 (13)	30 (10)
Major bleeding	3 (2)	9 (3)
Cessation of treatment	2 (1)	0 (0)
Death of any cause		
Within 1 year	8 (5)	0 (0)
Cumulative^a^	11 (7)	3 (1)
Recurrent VTE	2 (1)	3 (1)
Intended duration of anticoagulant therapy		
3 months	5 (3)	6 (2)
6 months	2 (1)	0 (0)
12 months	38 (24)	52 (17)
Indefinite	55 (34)	42 (14)

*CDT* catheter-directed thrombolysis, *USCDT* ultrasound-assisted CDT, *IQR* interquartile range (25–75th percentile), *VTE* venous thromboembolic event

* *P* < 0.05

^a^Including deaths within the first year

Major non-fatal bleeding occurred in three patients in the USCDT group and in two patients in the CDT group (9 vs. 3 %). There were seven patients (11 %) in the CDT group and one patient (3 %) in the USCDT group who died from any cause during follow-up. In both treatment groups, one patient developed treatment-related recurrent thrombotic event.

Forty-seven of 62 CDT patients (75 %) and 21 of 33 of USCDT patients (64 %) attended the follow-up visit. Table 3 displays long-term follow-up outcomes. Median time to follow-up was 89 months in the CDT group (range 15–141) and 34 in the USCDT group (range 17–51). Twice as much patients were still on anticoagulation in the CDT group than in the USCDT group (70 vs. 33 %, *P* < 0.05). Incidence of recurrent events and use of compression stockings (data not shown) was similar between groups.

**Table 3:** Long-term outcomes after (ultrasound-assisted) catheter-directed thrombolysis in 68 cases of iliofemoral or inferior vena cava deep vein thrombosis

	CDT (*n* = 47)	USCDT (*n* = 21)
	% (*n*)	% (*n*)
Median follow-up, months (range)	89 (15–141)	34 (17–51)
Anticoagulant therapy		
On therapy at follow-up	70 (33)	33 (7)*
Median time, months (range)	12 (6–75)	12 (3–24)
Recurrent VTE	2 (1)	0(0)
Post-thrombotic syndrome		
None	45 (21)	48 (10)
Mild	32 (15)	28 (6)
Moderate	13 (6)	19 (4)
Severe	10 (5)	5 (1)
SF-36 (mean ± SD)		
Physical subscale	40 ± 13	45 ± 12
Mental subscale	53 ± 9	48 ± 12
VEINES-QOL/Sym (mean ± SD)		
QOL subscale	50 ± 7	51 ± 6
Sym subscale	48 ± 7	50 ± 7
Patency at venography		
Attendance (n)	38	17
Open	79 (30)	76 (13)
Stenosis	8 (3)	0 (0)
Occluded	13 (5)	24 (4)

*CDT* catheter-directed thrombolysis, *USCDT* ultrasound-assisted CDT, *VTE* venous thromboembolic event, *SD* standard deviation

* *P* < 0.05

Fifty-five percent in the USCDT and 52 % in the CDT group developed PTS (Table 3). We observed non-significant lower prevalence of *severe* PTS in the USCDT group (5 vs. 10 %). There were no significant differences between groups regarding quality of life scores (Table 3). Vascular patency after long-term follow-up did not differ between groups [complete patency 79 (CDT) vs. 76 % (USCDT)].

## Discussion

We found that USCDT was associated with a higher proportion of patients requiring short treatment (<48 h), shorter duration of the hospital stay, and less intravenous stenting of residual thrombosis after thrombolysis. Short-term vessel patency and bleeding complications did not differ between groups. Likewise, long-term vessel patency, prevalence of PTS, and quality of life scores were essentially similar. Thus, we were not able to confirm the previous findings of improved vessel patency [13–15, 23] or the decreased amount of thrombolytic agent used for USCDT. Our findings suggest that USCDT does not have any apparent clinical benefits over CDT alone.

Our findings are in agreement with the randomized study by Engelberger et al. [16], who neither found any differences in thrombus load when comparing CDT with USCDT nor in vessel patency and incidence of PTS 3 months after treatment. These findings rejected their hypothesis [24, 25], assuming that USCDT would improve the reduction of thrombus load directly after diagnosis of an acute DVT, leading to a lower incidence of PTS in the future. Similarly, Baker et al. [15] did not find a difference between USCDT (*n* = 64) and CDT (*n* = 19) in thrombus resolution directly after the procedure. A possible explanation for the lack of differences between treatment modalities may be too low power of the ultrasound device used [16]. The EKOS MACH4 device that we used has an ultrasound frequency of 2.05–2.35 MHz and 0.5 W power per transducer. Experimental studies have suggested a thrombolysis optimum of around 2.2 MHz with a higher transmitted power (1, 2, 4, or 8 W per square centimeter) [14, 26]. Lysis of human clots has been shown to increase significantly when ultrasound was applied at 1.0 or 1.5 W [27]. The lower power may partly explain the lack of beneficial impact of USCDT treatment.

Our study has some advantages. Most importantly, our study had longer follow-up of patients (minimally 15 months in both groups) than other studies (minimal 3 or 6 months). This allowed us to assess the prevalence of PTS at a time point when most patients are near reaching a stable level of their PTS [1], i.e., looking at real long-term sequels of DVT. Second, we performed a new venography in most attendees after the follow-up, allowing us to associate a subjective outcome as PTS with vessel patency.

Due to the non-randomized design, confounding might have influenced our results. However, a systematic difference is unlikely as the patient and pretreatment characteristics were essentially similar between treatment modalities *and* between patients admitted for therapy before and after 2009. Second, medical specialists as well as treatment protocols remained the same over the years, thereby reducing the possibility of confounding. However, we cannot exclude the influence of minor unknown confounders that occurred because of time passing. Our study reflects a real life setting and is therefore less prone to selection bias compared to a randomized controlled trial, where participants tend to be younger and healthier compared to the population they are recruited from. An alternative explanation for our finding of a reduced treatment time in the USCDT group might be a learning effect: medical specialists are assumed to be more experienced over time with the techniques and logistics of an intravenous catheter-directed thrombolytic procedure in DVT patients. Shortening of the hospital stay in the USCDT group might reflect a change in general health care recommendations rather than the effect of adding ultrasound to the treatment. This might also be true for our finding that in the USCDT group less intravenous stenting was used, thus reflecting not a true effect of the treatment but more a general growth in insights in interventional radiology. Another minor drawback of our study is that the adjudicators of the Villalta Scale and quality of life were not blinded for treatment, but this generally increases the chance of a type I error and not that of type II. Finally, the prevalence of PTS (55 % in the CDT group and 52 % in the USCDT group) is somewhat higher than that reported in the literature [1]. This might be due to our long follow-up in both arms, and also by the fact that we included only patients with an iliofemoral or more proximal DVT, who are known to be more prone to develop PTS [1]. Selection of participants with PTS at the follow-up clinical examination might also contribute to the high prevalence of PTS.

In conclusion, we found that USCDT leads to a higher proportion of patients that needed short duration of thrombolysis, shorter hospital stay, and less frequent intravenous stenting. In accordance with previous studies, we showed that USCDT was not superior to CDT with regard to short- and long-term vessel patency, the long-term prevalence of PTS, and quality of life. Our findings suggest that USCDT does not have any apparent clinical benefits over CDT alone. However, due to limited available data, a large randomized trial comparing USCDT and CDT with long-term follow-up is warranted.
